# White matter changes in Parkinson’s disease

**DOI:** 10.1038/s41531-023-00592-z

**Published:** 2023-10-31

**Authors:** Kai Yang, Zhengqi Wu, Jie Long, Wenxin Li, Xi Wang, Ning Hu, Xinyue Zhao, Taolei Sun

**Affiliations:** 1 School of Chemistry, Chemical Engineering and Life Science, Wuhan University of Technology, 122 Luoshi Road, Wuhan, 430070 People’s Republic of China; 2 State Key Laboratory of Advanced Technology for Materials Synthesis and Processing, Wuhan University of Technology, 122 Luoshi Road, Wuhan, 430070 People’s Republic of China

**Keywords:** Parkinson's disease, Parkinson's disease

## Abstract

Parkinson’s disease (PD) is the second most common neurodegenerative disease after Alzheimer’s disease (AD). It is characterized by a progressive loss of dopaminergic neurons in the substantia nigra pars compacta (SNc) and the formation of Lewy bodies (LBs). Although PD is primarily considered a gray matter (GM) disease, alterations in white matter (WM) have gained increasing attention in PD research recently. Here we review evidence collected by magnetic resonance imaging (MRI) techniques which indicate WM abnormalities in PD, and discuss the correlations between WM changes and specific PD symptoms. Then we summarize transcriptome and genome studies showing the changes of oligodendrocyte (OLs)/myelin in PD. We conclude that WM abnormalities caused by the changes of myelin/OLs might be important for PD pathology, which could be potential targets for PD treatment.

## Introduction

Parkinson’s disease (PD) is the second most common neurodegenerative disease after Alzheimer’s disease (AD). It is characterized by a progressive loss of dopaminergic neurons in the substantia nigra pars compacta (SNc) and the formation of Lewy bodies (LBs)^1^. LBs are α-synuclein-rich intracellular inclusions, an essential pathological hallmark for PD and several other neurodegenerative diseases^2^. PD patients show several cardinal motor deficits, including bradykinesia, rigidity, postural instability and resting tremor^1^. They are also associated with many non-motor symptoms, such as depression, sensory abnormalities, sleep disorder and cognitive impairment^3^. Cognitive impairment is particularly common in PD, varying from mild cognitive impairment (MCI) to dementia. Dementia occurs in the advanced stage of the disease while MCI is common in the early disease phase^4^.

Currently, dopamine replacement therapy using L-3,4-dihydroxyphenylalanine (L-DOPA) is very common for PD patients, but most patients develop L-DOPA induced dyskinesia (LID) after long-term administration^5,6^. Other dopaminergic therapies, including dopamine agonists, monoamine oxidase B (MAO-B) inhibitors and catechol-O-methyltransferase (COMT) inhibitors are only partially effective^7^. Therefore, a new therapeutic approach is urgently required.

The CNS tissues, including the brain and spinal cord, can be divided into gray (GM) and white matter (WM) based on their distinctive coloring. GM includes the cell bodies and dendrites of the neurons while WM consists of the myelinated axons. The white coloring of WM is caused by the presence of myelin, a fatty sheath that wraps around most axons in the WM. Myelin is produced by oligodendrocytes (OLs). The myelination of axon plays a fundamental role in action potential propagation during synaptic transmission. In addition, it supports axonal integrity by providing metabolic and trophic support to the neurons^8^.

Although PD is primarily considered a GM disease, alterations in WM have gained increasing attention in PD research recently. In PD patients, α-synuclein proteins are misfolded and aggregated in neurons. While in patients with multiple system atrophy (MSA), α-synuclein predominantly accumulates in OLs and forms glial cytoplasmic inclusions (GCIs)^9^. However, this distinction is not absolute. Recent studies have identified the existence of GCI-like structures in OLs of PD patients carrying α-synuclein gene mutations (G51D and A53E)^10,11^. In addition, a PD patient with atypical clinical features exhibits demyelinating lesions in several brain areas including the right parietal, occipital and entorhinal cortex^12^.

Consistently, biochemical studies have detected changes of OLs in PD. It has been shown that the levels of CNPase protein are reduced significantly in the striatum after 1-methyl-4-phenyl-1,2,3,6-tetrahydropyridine (MPTP) injection in mice, suggesting that OLs in the striatum are damaged^13^. In contrast, in both mice and nonhuman primates, MPTP treatment induces oligodendrogliosis demonstrated by an increase in OL cell number and average size, which significantly correlates with the loss of dopaminergic neurons in SNc^14^. It is proposed that these increased numbers of OLs might be caused by the compensation for disease-related myelin loss.

Changes of other OL functions have also been revealed in PD. For example, although there is no neuron reduction in motor cortex in the early phases of PD, structural changes occur. During PD progression, α-synuclein accumulates in the WM of the motor cortex, resulting in the reduction of myelin proteins as well as an increase in OL lineage cell (OLC) density and the size of mature OLs^15^. Furthermore, when O4+ OLCs are derived from PD patient-induced stem cells (iPSCs), their ability to mature into myelinating OLs is impaired. Instead, they adopt an antigen-presenting phenotype. This antigen-presenting phenotype of OLCs can also be induced by a fibrillar α-synuclein variant (p.A53T). But in this study, how this antigen-presenting OLC contributes to PD remains unstudied^16^.

Here we review evidence collected by magnetic resonance imaging (MRI) techniques indicating WM abnormalities in PD, and discuss the correlation between WM changes and specific PD symptoms. Then we present transcriptome and genome studies showing the changes of OLs/myelin in PD. We conclude that WM abnormalities caused by the changes of myelin/OLs might be important for PD pathology, which could be potential targets for PD treatment.

## Evidence from imaging studies

### WM hyperintensities (WMHs)

WMHs, one of the indicators of cerebral small vessel disease (CSVD), are commonly found on brain MRI in PD patients^17,18^. These signals usually appear in periventricular and deep WM when visualized by T2-weighted MRI or fluid-attenuated inversion recovery (FLAIR) sequences. WMHs are closely linked to motor symptoms in PD patients^19^, results show that nondopaminergic subcortical pathways are involved in the pathogenesis of these PD features^20^. Nonetheless, this conclusion needs further confirmation because some studies fail to report this association in PD^21^. In addition, PD dementia (PDD) has more severe WMH than do PD with normal cognition (PD-NC) and controls^22,23^, but the association between WMH and cognitive impairment in PD is still under debate^24,25^ (Tables 1 and 2).

**Table 1:** Summary and description of WMH, VBM, dMRI and myelin imaging metrics.

Modality	Metric name	Abbreviation	Description
WMH	WMH score	WMH score	Score of WMH
VBM	MNI coordinates	MNI coordinates	Montreal Neurological Institute coordinates of local maxima
	Cluster size	Cluster size	Voxel numbers with a significant difference in volume between groups
	Max T value	Max T value	The voxel maximum in the cluster
DTI	Fractional anisotropy	FA	The directionality of water molecule
	Axial diffusivity	AD	Microstructural directions in parallel to the WM tracts
	Radial diffusivity	RD	Microstructural directions in perpendicularly to the WM tracts
	Mean diffusivity	MD	The average of AD and RD
DKI	Axial Kurtosis	AK	The tissue complexity parallel to the direction of water diffusion
	Radial Kurtosis	RK	The tissue complexity perpendicular to the direction of water diffusion
	Mean Kurtosis	MK	Overall microstructural complexity of the brain
NODDI	Neurite density index	NDI	Intracellular volume fraction of water in neurites
	Orientation dispersion index	ODI	Tract complexity across individual tracts
	Isotropic volume fraction	IVF	Extracellular unhindered water diffusion
MWI	Myelin water fraction	MWF	The ratio of the area in the T2 distribution from MW to the area of entire T2 distribution
MTI	Magnetization transfer ratio	MTR	Magnetization transfer between protons bound to myelin and those bound to water
QSM	Mean magnetic susceptibility	MMS	The mean of magnetic susceptibility

**Table 2:** An overview of the literature about WM changes in PD using WMH and VBM.

Study	Objective studied	WMH (WM)	VBM (WM)
Lee et al.^19^	PD patients	Deep WMHs are associated with bradykinesia, periventricular hyperintensities are associated with bradykinesia and axial symptoms in PD patients	
Wan et al.^20^	PIGD	A close association between axial motor impairments and DWMH of the frontal and occipital lobes	
Marshall et al.^23^	PDD	PDD > Controls: periventricular and deep WM	
Dalaker et al.^24^	PD patients	PD = Controls: total WMH volume	
Nyatega et al.^41^	PD patients		PD < Controls: right middle cingulate, left lingual gyrus, right calcarine, and left inferior occipital gyrus
Wen et al.^46^	PD-MCI		PD-MCI < PD: bilateral Middle and Medial frontal gyrus
Kostic et al.^44^	PD with depression		PD with depression < PD without Depression: right AC bundle and inferior orbitofrontal (OF) region
Shigemoto et al.^45^	MSA-P		MSA-P < Controls: bilateral globus pallidi, external capsules extending to the midbrain, right subcortical to precentral area through internal capsule, the pons, bilateral middle cerebellar peduncles and left cerebellum.
Rektor et al.^47^	PD with normal cognition		PD with normal cognition = Controls
Zheng et al.^48^	PD patients		PD = Controls

It has been proposed that WMHs are caused by the reduction of myelin due to Wallerian degeneration^26^, but they are not the ideal indicators for WM changes, other factors such as blood–brain barrier (BBB) impairment, damage to the microvascular structure and the dysfunction of cerebrovascular autoregulation also contribute to them^27^.

### Voxel-based morphometry (VBM)

VBM is a method of statistically analyzing morphological changes in the brain^28^. It detects volume differences between groups by performing statistical tests across all voxels in T1-weighted volumetric MRI images. VBM has been widely used in various diseases including AD and multiple sclerosis (MS)^29,30^. In addition, regional brain changes are unique in different diseases, so the region-specific changes in the brain can be used as an indicator of the disease.

In the past few years, VBM has been widely used to study GM atrophy in the brain of PD patients^31–37^. Many MRI studies have detected GM atrophy in PDD^38–41^, but few studies have demonstrated that there is no GM atrophy in PD patients with mild cognitive impairment (PD-MCI)^42,43^.

WM trophy in PD patients has also been detected using VBM. Many studies have observed the volume reduction of WM in PD^41,44–46^. In comparison to PD patients without depression, PD patients with depression exhibit a more severe WM loss in the right frontal lobe, including the anterior cingulate bundle (ACB) and the inferior orbitofrontal (IOF) region. In addition, the severity of depression is significantly correlated with the WM loss in the right IOF region^44^. Another study showed that PD-MCI demonstrate longitudinal reduction in WM volume, especially in the frontal areas^46^. Furthermore, in comparison to controls, PD patients show reduced WM volumes in the right middle cingulate (RMC), left lingual gyrus (LLG) and left inferior occipital gyrus (LIOG)^41^. Moreover, in MSA with predominant parkinsonism (MSA-P), WM loss is detected in bilateral globus pallidi (GP), external capsules (EC) extending to the midbrain and right subcortical to precentral area^45^. Inconsistently, several studies observed no volume reduction of WM in PD^47,48^ (Tables 1 and 2).

However, the measurement of WM volume loss does not indicate disease stage and it is also not sensitive to changes in microstructure. The reductions in WM volume could be caused by either the decrease in axon numbers or the reduction of axon myelination.

### Diffusive MRI (dMRI)

dMRI consists of several techniques which detect microstructural integrity non-invasively via the diffusion of water molecules in the brain. Diffusion tensor imaging (DTI), the most common dMRI model, provides more in-depth information about WM organization at the microstructural level. It estimates brain microstructures by measuring both the direction and magnitude of water molecule diffusion in the brain^49^. The most commonly used parameters in DTI are fractional anisotropy (FA), mean diffusivity (MD), axial diffusivity (AD) and radial diffusivity (RD) (Table 1)^50^. FA represents the directionality of water molecules, a decrease of FA indicating the impairment of WM microstructural integrity. AD and RD represent microstructural directions parallel and perpendicular to the WM tracts respectively^51^. An increase of RD indicates demyelination^52^, whereas decreased AD represents axonal injury^53^. MD derives from the average of AD and RD, high MD indicating broad cellular damages. Usually, structurally intact WM has a high FA and low MD, whereas damaged WM has a low FA and high MD.

Although the DTI technique has been widely used in WM imaging, it has several limitations: (1) DTI modeling assumes that the probability of water diffusion follows the Gaussian distribution, but in the brain water diffusion does not fit this assumption^54^. (2) DTI parameters have been widely used to indicate specific pathological changes, but these correlations are not accurate^55^. For example, either demyelination or axonal loss could result in decreased FA and increased MD. In addition, although some studies have shown that AD and RD can indicate axonal loss and demyelination respectively^56^, recently these correlations have been questioned by other studies^57^. (3) DTI assumes the voxel is a single-tissue compartment, but in the brain the existence of cerebrospinal fluid (CSF) goes against this assumption^58^. (4) DTI is especially useful in evaluating the WM microstructures because WM has a significant degree of anisotropy.

To address these limitations of DTI, advanced dMRI techniques, including diffusional kurtosis imaging (DKI) and neurite orientation dispersion and density imaging (NODDI), have been developed. In contrast to DTI, DKI has the ability to assess non-Gaussian diffusion of water molecules. Kurtosis reflects the degree of hindrance to the diffusion of water molecules in the brain^59,60^. Usually, high kurtosis indicates great structural integrity in tissue, whereas a decrease in kurtosis reflects neuronal loss^61^. DKI provides not only DTI metrics (AD, RD, MD and FA) but also kurtosis metrics (axial kurtosis (AK), radial kurtosis (RK) and mean kurtosis (MK)) (Table 1). MK reflects the overall microstructural complexity of the brain, whereas AK and RK represent the tissue complexity parallel and perpendicular to the direction of water diffusion respectively. In addition. kurtosis parameters can be applied in both isotropic and anisotropic environments^60^, so they are useful in evaluating microstructural changes in both GM and WM.

Another MRI technique, NODDI, proposes that water molecules in the brain are confined within three separate compartments. (1) The intracellular compartment, (2) the extracellular compartment, and (3) a CSF compartment. The microstructure of the dendrites and axons is estimated by quantifying the neurite density (NDI, indexed by intracellular volume fraction (ICVF)), orientation dispersion index (ODI) and isotropic volume fraction (ISOVF) (Table 1). This technique has several advantages: it can delineate WM from GM, usually, WM exhibits a higher NDI and lower ODI, while GM displays a lower NDI and higher ODI^62^. In addition, it has the ability to differentiate between different GM structures^63^.

### DTI

PD patients exhibit a variety of symptoms, such as motor symptoms including postural instability and gait disorder (PIGD) and freezing of gait (FOG) as well as non-motor symptoms, including cognitive impairment and depression. A complete understanding of the underlying mechanisms in PD symptoms will potentially result in improved symptom management. Therefore, studying the correlations between specific PD symptoms and DTI metrics might help us understand the neuropathological underpinnings of PD symptomatology.

### DTI correlates of motor dysfunction

Many studies have investigated the association between DTI changes and PD motor symptoms. PD can be divided into two subtypes: tremor dominant (TD) and PIGD. These two subtypes demonstrate differences in not only their dominant motor symptoms, but also non-motor symptoms. Compared to TD patients, PIGD patients have more severe cognitive impairment and faster disease progression, Traditionally, studies in PIGD patients have mainly focused on the pathophysiologic changes in the nigrostriatal and extrapyramidal pathways. Recently, there is increasing evidence showing that both cortical and subcortical regions are involved in PIGD^64,65^.

It has been shown that the nucleus basalis of Meynert (NBM)-WM tracts are associated with PD-PIGD. FA in the frontal NBM-WM tracts is significantly lower in PD-PIGD than PD-TD. In addition, the severity of motor symptoms is significantly correlated with lower FA and higher MD in these tracts in PD-PIGD^64^. Furthermore, the periventricular fibers are also linked to PD-PIGD. The superior longitudinal fasciculus (SLF), inferior longitudinal fasciculus (ILF), genu the corpus callosum (GCC) and body of the corpus callosum (BCC) are more affected in PIGD than in PD and controls. DTI measures in the SLF and GCC fibers are correlated with clinical gait severity^65^. Additionally, WM alterations between PD-PIGD and PD-TD in the early stages have been compared. PD-TD patients demonstrate greater FA and reduced RD and AD compared with controls, suggesting greater WM integrity and less axonal degeneration and demyelination, which is caused by the neural reorganization to compensate for the loss of dopaminergic neurons in TD. In contrast, PD-PIGD patients exhibit more WM degradation, which is indicated by decreased FA but increased RD and AD, compared with TD patients^66^ (Table 3).

**Table 3:** An overview of the literature about WM changes in PD using DTI.

Study	Objective studied	FA reductions	MD (AD and RD) increases
Motor correlates			
Nazmuddin et al.^64^	PIGD	PIGD < TD: bilaterally at the proximal portion of the medial NBM tract and the frontotemporal portal of the lateral NBM-WM tract; PD, PIGD < controls: fronto-lateral NBM-WM tract of the left hemisphere	
Tan et al.^65^	PIGD	PD, PIGD < controls: inferior longitudinal fasciculus, inferior fronto-occipital fasciculus, anterior thalamic radiation	PD, PIGD > controls: superior longitudinal fasciculus, inferior longitudinal fasciculus, inferior fronto-occipital fasciculus, anterior thalamic radiation, and body of the corpus callosum; PIGD > PD: superior longitudinal fasciculus, genu of the corpus callosum, and body of the corpus callosum
Wen et al.^66^	PIGD	PIGD < TD: bilateral inferior longitudinal fasciculi, right posterior thalamic radiation, right superior and posterior corona radiata	PIGD > TD: bilateral inferior longitudinal fasciculi, bilateral posterior thalamic radiation, bilateral anterior and posterior corona radiata, left uncinate fasciculus
Canu et al.^67^	FOG	The primary motor, premotor, prefrontal, orbitofrontal, and inferior parietal cortices, cingulum, and superior longitudinal fasciculus bilaterally, and of the cerebral peduncles, corpus callosum (genu, anterior body, and splenium) and temporo-occipital WM tracts	The primary motor, premotor, prefrontal, orbitofrontal, and inferior parietal cortices, cingulum, and superior longitudinal fasciculus bilaterally, and of the cerebral peduncles, left corticospinal tract and thalamic radiations, external capsule, bilaterally, and cerebellum (right lobule VIII, and lobule IX, bilaterally)
Fling et al.^68^	FOG	PD-FOG vs. PD and Controls: PUT, internal GPi, cingulate, Thal, precentral and postcentral gyrus, superior and middle frontal gyrus, SMA, pre-SMA and bilateral cerebellar locomotor regions PPN fiber tract in the right hemisphere	
Peterson et al.^69^	FOG	Greater quantity in PPN tract in the right hemisphere; Correlation in FOG: lateralized PPN connectivity and larger dual task interference, stride length during normal and dual task walking	
Vercruysse et al.^71^	FOG	Left (lobule VI) and right (Crus I, lobule VIIIb) hemisphere of the cerebellum, left temporal part of the SLF; connections between the left caudate nucleus and ACC and orbitofrontal cortex, between the left middle CP and PPN	Right anterior part of the capsula interna and corona radiata, the superior frontal cortex, and a left-hemispheric cluster in cerebellum Crus II; connecting the caudate, PUT, GP, and STN to frontal (ACC, superior and orbitofrontal cortex), motor (pre-SMA, SMA) and sensory (S1 and S2) cortical regions predominantly in the left hemisphere
Youn et al.^72^	FOG	Bilateral PPN	Basal ganglia, Thal and cerebellum (in PPN tract); bilateral PPN
Cognitive correlates			
Agosta et al.^73^	MCI	PD-MCI vs. PD: bilateral anterior and superior corona radiata, CC genu and body anterior inferior IFOF/uncinate, anterior SLF; PD-MCI vs. Controls: bilateral anterior and superior corona radiata, CC body, anterior SLF	
Kamagata et al.^74^	PDD	PD < Controls: anterior cingulate bundle; PDD < Controls: anterior and posterior cingulate bundle	PD > Controls: anterior cingulate bundle
Kamagata et al.^75^	PDD	PDD < PD: anterior part of the IF-OF and in part of the genu of CC; PDD < Controls: SLF, ILF, IFOF, UF, cingulum, anterior limb of internal capsule, SN (all bilateral)	PDD > PD: anterior part of the IF-OF and in part of the genu of CC; PDD > Controls: SLF, ILF, IF-OF, UF, cingulum, anterior and posterior limb of internal capsule, SN (all bilateral)
Bledsoe et al.^76^	PDD		PD > Controls: callosal segment 1,2 and 3; PDD > PD-NC: callosal segment 1 and 2; PDD > PD-MCI: callosal segment 1
Chondrogiorgi et al.^77^	PDD	PDD < PD: the body of corpus callosum, bilaterally in superior corona radiata and cingulum (cingulate gyrus) and on the left side in anterior corona radiata, inferior fronto-occipital fasciculus (IFF), uncinate fasciculus, anterior thalamic radiation (ATR) and forceps minor	
Mood correlates			
Surdhar et al.^83^	Depression		
Prange et al.^80^	Depression	PDD < Controls: anterior interhemispheric connections and limbic association tracts	
Lacey et al.^82^	Depression		
Surdhar et al.^83^	Depression		

DTI changes have also been associated with FOG, which is a sudden and transient gait disturbance where the patient has no ability to move forward despite the intention to walk. Several studies have shown that structural connections in subcortical brain regions including the pedunculopontine nucleus (PPN) are involved in PD-FOG^67–72^. Abnormal connectivity between the PPN and a number of cortical and subcortical regions has been demonstrated^70^. In addition, several DTI studies have confirmed that PPN connectivity is impaired in PD-FOG compared to PD without FOG (PD-nFOG)^67–69,71,72^. Long associative WM bundles are impaired in PD-FOG as well. Compared with PD-nFOG, cortico-cortical WM tracts, the GCC and the splenium of the corpus callosum (SCC) are damaged in PD-FOG^67^. Furthermore, WM changes correlated with FOG severity in most of the DTI studies^67,70–72^ (Table 3).

### DTI correlates of cognition impairment

Cognitive dysfunction is one of the most common non-motor symptoms for PD patients. WM tracts are important for efficient cognitive processing and changes in WM anisotropy have consistently been linked to cognitive deficiencies. It has been shown that WM tracts are damaged in PD patients with cognitive impairments^73–75^.

Cognitive decline in PD can be caused by the disruption of corpus callosum (CC), which is a critical structure for interhemispheric information transfer and plays an essential role in cognitive function^76,77^. One study has shown that multifocal microstructural changes of WM accompany the transition of cognitive state from normal to impaired in PD patients. Compared with PD without dementia, FA values in the CC, corona radiata and cingulum are significantly reduced in PDD^77^. In addition, when the entire CC is segmented into three sub-regions, PDD patients exhibit an increase of diffusivity metrics except FA in the most anterior callosal (AC) segment compared to controls, PD-NC and PD-MCI^76^ (Table 3).

### DTI correlates of depression

Some DTI studies have demonstrated that depressed PD patients display reduced FA in many WM tracts, including the left uncinate fasciculus (UF)^78–80^. The UF is a WM tract that connects the frontal lobe and subcortical structures. Disruption of the UF impairs the communication between the frontal lobe and subcortical regions, which impedes top-down controls. Then the lower regions including the amygdala will demonstrate unmodulated activities, which induces emotional disturbances^81^. However, in other studies no significant differences were observed in the WM tracts between PD patients with and without depression^82,83^ (Table 3). Thus, further studies are required to understand the association of WM integrity with depressed PD.

### DKI

DKI is more sensitive to WM changes than DTI and it can reveal more extensive microstructural changes in PD patients. In PD patients, DKI can detect decreased MK in the frontal, occipital, parietal and right temporal WM, whereas DTI can only display decreased FA in the frontal WM^84^. In addition, although both MK and FA are decreased in the anterior cingulum (AC) fiber of PD patients, only MK exhibits the best diagnostic performance. Importantly, AC shows pathological changes at an early stage and MK in this region can be used as a diagnostic biomarker for PD^85^. In contrast, MK is increased in several WM areas of PD animal models^86–88^ (Table 4). This discrepancy might be caused by the accumulation of α-synuclein in the transgenic PD animal model used in this study. The accumulation of α-synuclein contributes to the increase of kurtosis values while loss of neurons in PD patients causes the opposite result.

**Table 4:** An overview of the literature about WM changes in PD using DKI and NODDI.

Study	Objective studied	DKI metrics	NODDI metrics
Kamagata et al.^85^	PD patients	PD < Controls (MK and FA): anterior and posterior cingulum	
Kamagata et al.^84^	PD patients	PD < Controls (FA): anterior part of the inferior fronto-occipital fasciculus (IFOF), anterior SLF, and anterior and superior corona radiata; in part of the posterior SLF and in part of the genu and body of the corpus callosum: PD < Controls (MK): the SLF and inferior longitudinal fasciculus; the IFOF; the uncinate fasciculus; and the anterior, posterior, and superior corona radiata	
Arab et al.^86^	METH-treated animals	PD > Controls (FA): primary somatosensory cortex, fornix and ventral nucleus of the lateral lemniscus; PD > Controls (RK): in ventral nucleus of the lateral lemniscus and some of the lateral thalamic nuclei; PD < Controls (MD): in cingulum, external capsule and some of the lateral thalamic nuclei; PD < Controls (AD): cingulate cortex and external capsule; PD < Controls (RD): cingulum, primary somatosensory cortex, external capsule, some of the lateral thalamic nuclei and ventral nucleus of the lateral lemniscus	
Khairnar et al.^88^	TNWT-61 mice	PD > Controls (MK): the anterior and posterior part of the anterior commissure, in the internal capsule, left habenular commissure, medial lemniscus, mammillothalamic tract, external capsule, and reticular formation; PD > Controls (AK): the right-sided external capsule; PD < Controls (AD): ventral hippocampal commissure, trajection from/to septofimbrial nucleus, corpus callosum, external capsule, and in the fornix	
Khairnar et al.^87^	TNWT-61 mice	PD > Controls (MK): bilaterally in the external and internal capsule, anterior commissure (and in the posterior nerve part), lateral stripe of the striatum, diagonal band around the lateral septal nucleus and around the thalamus, mammillothalamic tract, fornix, cerebral peduncle, medial lemniscus, cingulum, reticular formation, medial longitudinal fasciculus, deep and intermediate white layers of the superior colliculus; corpus callosum, left-sided medial forebrain bundle, and brachium of the superior colliculus	
Andica et al.^89^	PD patients with neurocognitive and psychiatric disorders		PD-woNCPs > PDwNCPs (ICVF): Bilateral CST, cingulum hippocampus, IFOF, ILF, SLF, SLF temporal part, retrolenticular part of internal capsule, SCR, PCR, PTR, sagittal stratum, external capsule, tapetum; left PLIC; right ATR, CCG, UF; forceps major, body and splenium of corpus callosum; PD-wNCPs < Controls: Bilateral ATR, CST, CCG, cingulum hippocampus, IFOF, ILF, SLF, UF, SLF temporal part, ALIC, PLIC, retrolenticular part of internal capsule, ACR, SCR, PCR, PTR, sagittal stratum, external capsule, SFOF, and tapetum; forceps major and minor, genu, body, and splenium of corpus callosum
Ogawa et al.^90^	PD patients with LID		PD wLID < PD woLID (ICVF): the external capsule, inferior fronto-occipital fasciculus, inferior longitudinal fasciculus, and uncinate fasciculus

### NODDI

NODDI is also superior over DTI in the detection of PD pathology. In PD patients with neurocognitive and psychiatric disorders (NCPs), ICVF is reduced in the WM when compared to those without these symptoms^89^. Furthermore, ICVF significantly contributes to the main effects of diagnosis^89^, thus can be used as a biomarker to detect microstructural changes in the WM which are related to NCPs in PD. In addition, WT microstructural alterations in PD patients are measured using advanced dMRI methods (DTI, DKI, and NODDI). It has been shown that PD with LID demonstrates fewer WM microstructural changes, especially in temporal lobe fibers, than does PD without LID^90^ (Table 4).

### Myelin imaging

In demyelinating diseases, myelin is damaged, leaving the axons vulnerable to stress. Protecting or even restoring myelin is a promising therapy for demyelination diseases. Myelin imaging provides a biomarker to evaluate the efficacy of therapy. In addition, myelin imaging can be used to monitor disease progression. Considering the important roles of myelination, there is a high demand for developing MRI techniques to image myelin. Several methods have been developed for myelin imaging using MRI:

1. Myelin water imaging (MWI), which detects the water pool between myelin bilayers directly. Usually, conventional MRI cannot be used to detect myelin damage directly; however, such information can be inferred indirectly by analyzing T2 relaxation of the water molecules in the tissue containing myelin. The water which is trapped between the myelin bilayers is called myelin water (MW). It experiences a fast decay rate and leads to a short T2, whereas mobile water pools including intra and extracellular water (IEW) and free water contribute to a long T2. These signals are superimposed to form a decay curve. Using non-negative least squares (NNLS), the decay curve can be decomposed into a distribution of T2 times. It has been proposed that MW contributes to the short T2 components. The myelin water fraction (MWF) is defined as the ratio of MW components to the total distribution, which can be used to indicate the myelin abundance in the brain (Table 1).
2. Magnetization transfer imaging (MTI), which measures magnetization transfer between protons bound to myelin and those bound to water. This method measures the transfer of magnetization exchange between protons bound to myelin and those bound to water^91^. In conventional MRI, protons bound to myelin have a short T2, while proton signals from mobile water have a long T2 and can be detected directly. After applying an off-resonance radio frequency (RF) pulse, the magnetization of bound protons is saturated. This saturation is then transferred to the protons of mobile water, which results in a decrease of both longitudinal magnetization and signal intensity. The extent of signal decrease induced by the magnetization transfer is assessed by the MT ratio (MTR), which is decreased during demyelination (Table 1).
3. Quantitative susceptibility mapping (QSM), which maps the spatial distribution of myelin sheath’s magnetic susceptibility. It can measure the spatial distribution of magnetic susceptibility in the brain. Magnetic susceptibility is an intrinsic property of tissue, representing the change in magnetization of a material in response to an external magnetic field. Biological tissues can be either paramagnetic or diamagnetic depending on their compositions and microstructure. In the brain, myelin is the most important diamagnetic substance while iron is the major contributor of paramagnetic susceptibility. Therefore, magnetic susceptibility is considerably affected by demyelination.

### MWI

A voxel-wise approach is used to compare the MWF between PD and control groups, it shows that myelin is altered in the frontal and temporal WM in PD^92^. Another study used MWF to investigate the alterations of myelin content in 20 different WM regions of interest (ROIs), although there are no significant differences in WM microstructural integrity between PD and controls using univariate tests, when partial least squares (PLS) is used, myelin changes are found to be negatively correlated with PD clinical scores^93^. In addition, in order to characterize PD symptoms, MWF, FA, and resting-state functional connectivity (FC) are used as multi-sequence MRI markers, myelin changes are found to be strongly linked to PD rigidity^94^ (Table 5). While MWF shows good sensitivity to myelin changes, other factors also influence the measurement of myelin in vivo including myelin debris. For example, in a rat sciatic nerve cut/crush injury model, MWF fails to differentiate among intact myelin, degenerating myelin and myelin debris^95^.

**Table 5:** An overview of the literature about WM changes in PD using myelin imaging.

Study	Objective studied	MWF	MTR	MMS
Dean III et al.^92^	PD patients	PD > Controls: the thalamic radiations and posterior limb of the internal capsule, right centrumsemiovale encompassing superior longitudinal fasciculus, genu of corpus callosumand selected frontal and temporal regions, the body of the corpus callosum		
Baumeister et al.^93^	PD patients with distinct clinical subtypes	Interhemispheric and long-range association fibers, projection fibers		
Morgen et al.^96^	PD patients		PD < Controls: the paraventricular WM	
Tambasco et al.^97^	PD patients		PD < Controls: the paraventricular WM	
Tambasco et al.^98^	Mild and advanced PD patients		Advanced PD < Controls: frontal white matter, substantia nigra pars reticulata, periventricular white matter and parietal white matter; mild PD < Controls: substantia nigra pars reticulata, periventricular white matter and parietal white matter	
Hanyu et al.^99^	PDD		PDD < Controls: frontal WM, genu of the corpus callosum	
Guan et al.^101^	PD patients			PD < Controls: bilateral frontal deep WM; PD > Controls: Body of corpus callosum, bilateral External capsule, bilateral superior and inferior longitudinal fasciculus

### MTI

Morgen et al. found that MTR is sensitive to PD progression in the paraventricular WM and neocortex in patients^96^. In the supratentorial WM, PD patients show reduced MTR compared to healthy controls^97^. Furthermore, in both mild and advanced PD patients, a significant MTR reduction occurs in both GM and WM. Moreover, the change of MTR in advanced PD demonstrates a wider distribution than that in mild PD^98^. Although these studies have detected a difference in MTR between PD and controls, one study failed to confirm it, showing that MTR is the same between PD and controls; only in PD patients with depression is the MTR in the subcortical WM significantly lower than that in controls^99^ (Table 5). Although this technique has been widely used in PD studies, it has some weakness. For example, it is very difficult to distinguish between myelin loss and inflammation by MTR. In both situations, the changes of signal intensities are similar^100^.

### QSM

It has been shown that QSM improves the detection sensitivity of regional ultrastructural changes in the WM of PD patients compared to DTI. In PD patients, QSM detects extensive WM changes including regions close to the frontal, parietal and temporal lobes, which are more widespread than those demonstrated by DTI^101^. In these studies, the susceptibility contrast in WM is mainly caused by the reduction of myelin content, not iron deposition^102,103^. Furthermore, changes in the WM are correlated with motor impairment and disease severity in PD patients^101^ (Tables 1 and 5).

## Evidence from transcriptome and genome studies

### Transcriptome studies

RNA-sequencing (RNA-seq) is a technique that measures the quantity and sequences of RNA in a tissue using next-generation sequencing (NGS). It can demonstrate which genes are activated in a cell, what their transcription levels are and at what time point they are turned on or shut off. Using this technique, the changes of gene transcription which relate to disease can be revealed. Another technique, the assay for transposase-accessible chromatin with sequencing (ATAC-Seq), is developed to study the changes of transcription factors (TFs) binding in diseases. It can uncover how chromatin is packed and which TFs affect gene expression during disease progression. After it was invented, ATAC-Seq has been widely used to study chromatin accessibility, binding of TFs and gene regulation in diseases.

The involvements of OLs/myelin in PD have been revealed by RNA-seq studies. In post-mortem brains from PD patients, OL-related genes are upregulated while the downregulated genes are enriched in dopaminergic neurons^104^. On the contrary, in the cingulate cortex of PD patients, myelin genes and the OL development pathways are downregulated, suggesting that myelination is impaired in the cingulate cortex in PD^105^. The downregulation of myelination pathways also occurs in the cingulate cortex of patients with dementia with LBs (DLBs)^105^. In addition, single-nuclei RNA-sequencing (snRNA-seq) reports the loss of OLs in post-mortem midbrain tissue from PD patients^106^.

Furthermore, in the cingulate cortex of PD patients, when RNA-seq data are combined with ATAC-seq data, several transcription factors related to myelin formation and gliogenesis, including Olig2, Sox8, Sox10, E2F1, and NKX6‐2, have been identified^105^. Through integrating cell type-specific reference data, OL-specific, myelin-associated genes have been identified in the frontal cortex of an α-synuclein overexpressing rat model and post-mortem samples from PD patients^107^.

Compared to heavily myelinated cortical neurons, the axons of dopaminergic neurons in SN are seldomly myelinated. Axon myelination can decrease the energy consumption for neurons to transmit signals. The more myelin, the less energy neurons require to send a signal. In addition, α-synuclein prefers to aggregate in unmyelinated axons. As a result, poorly myelinated dopamine neurons are more susceptible to external stressors and more vulnerable to degeneration than are fully myelinated cortical neurons. It also explains why OL-related genes in SN are more active because of the compensation in PD.

Up to now, no molecular differences between cortical regions and SN have been identified. The fraction of PD genetic risk mapping to cortical excitatory neurons is similar to that mapped to SN dopaminergic neurons. It has been proposed that distinct vulnerabilities between cortical regions and SN might be caused by their different metabolic requirements and local environments^104^.

### Genome studies

Genome-wide association study (GWAS) is a technique which identifies genetic markers associated with a disease by scanning the genomes from people with this disease. It can help us understand how genes contribute to the disease and develop better therapeutics. GWAS can uncover both copy-number variants and sequence variations in the human genome. Although in GWAS the most commonly studied genetic variants are single-nucleotide polymorphisms (SNPs), it also reveals the disease-associated DNA risk loci, which are the blocks of correlated SNPs showing a statistically significant association with diseases.

Genome studies have also shown that OL-related genes are associated with PD pathogenesis. Fine-mapping of PD GWAS loci has revealed four consensus SNPs in most known PD-associated loci, one of which is muscle blind-like protein 2 (MBNL2) enhancer in OLs^108^. SnRNA-seq of human SN tissue has shown that OL-specific differentially expressed genes are associated with PD genetic risks^109^. Recently, a significant association between the myelin-associated OL basic protein (MOBP) rs616147 variant and PD has been identified^110^. MOBP is a protein widely expressed in LBs of PD patients and important for structural maintenance of the myelin sheath in the CNS^111^.

## Conclusion

In the present review, we discussed evidence of WM changes in PD. Although PD has been tightly associated with nigral GM degeneration, accumulating evidence suggest that WM alternations also occur in PD, these changes possibly appearing even before the loss of dopaminergic neurons in the SNc^47,104^. It is possible that WM dysfunctions are directly responsible for some clinical symptoms of PD. Furthermore, the successful identification of early changes in the WM microstructure for PD patients is very important, since they can be used as markers for both early screening of PD patients and the quick evaluation of drug therapy^112^.

### Reporting summary

Further information on research design is available in the Nature Research Reporting Summary linked to this article.

### Supplementary information

reporting summary
